# Supplementary material for: Chronic N-Acetylcysteine Treatment Prevents Amphetamine-Induced Hyperactivity in Heterozygous Disc1 Mutant Mice, a Putative Prodromal Schizophrenia Animal Model
Source: Int J Mol Sci. 2022 Aug 20;23(16):9419. doi: 10.3390/ijms23169419 (PMC9408838; doi:10.3390/ijms23169419)
Supplement: Supplementary file 1 [file ijms-23-09419-s001.zip › ijms-1854313-supplementary.pdf]

Supplementary Table S1. Antibodies used in this study.

	Name	Application and Dilution	Catalog number and Company
Primary antibodies	Mouse anti-TH	WB; 1:1000	T1299; Sigma-Aldrich, St. Louis, MO, USA
	Rabbit anti-DAT	WB; 1:1000	ab11146; Abcam, Cambridge, UK
	Mouse anti-D1R	WB; 1:200	sc-33660; Santa Cruz Biotech, Santa Cruz, CA, USA
	Mouse anti-D2R	WB; 1:200	sc-5303; Santa Cruz Biotech, Santa Cruz, CA, USA
	Rabbit anti-PDE4B	WB; 1:1000	sc-25812; Santa Cruz Biotech, Santa Cruz, CA, USA
	Rabbit anti-CREB	WB; 1:1000	Mab 9197; Cell Signaling Danvers, MA, USA
	Rabbit anti-pCREB	WB; 1:1000	Mab 9198; Cell Signaling Danvers, MA, USA
	Mouse anti-GSK3α/β	WB; 1:1000	sc-7291; Santa Cruz Biotech, Santa Cruz, CA, USA
	Rabbit anti-GAPDH	WB; 1:5000	GeneTex, Irvine, CA, USA
	Mouse anti-PV	IHC: 1:1000	P3088; Sigma-Aldrich, St. Louis, MO, USA
Secondary antibodies	HRP-conjugated goat anti-mouse IgG	WB; 1:2000	The Jackson ImmunoResearch Laboratories, West Grove, PA, USA
	HRP-conjugated goat anti-rabbit IgG	WB; 1:2000	The Jackson ImmunoResearch Laboratories, West Grove, PA, USA
	Biotin-conjugated goat anti-mouse IgG	IHC; 1:1000	The Jackson ImmunoResearch Laboratories, West Grove, PA, USA

IHC: Immunohistochemistry; WB: Western blot.
